# Implementation of audiovisual recording in the operating room: a nationwide survey of stakeholder perspectives in France

**DOI:** 10.1186/s13037-025-00467-7

**Published:** 2026-02-03

**Authors:** Côme Slosse, Arnaud Allemang--Trivalle, Nicolas El Haïk-Wagner, Amandine Luc, Elodie Jeanbert, Eric Vibert, Hervé Bouaziz

**Affiliations:** 1 Department of Anesthesiology and Intensive Care, University Hospital of Nancy, Nancy, France; 2 Inserm IADI U1254, University of Lorraine, Nancy, France; 3 Haptic Intelligence Department, Max Planck Institute for Intelligent Systems, Heisenbergstraße 3, 70569 Stuttgart, Germany; 4 Department of Computer Science, IMT Atlantique, Lab-STICC, UMR CNRS 6285, Brest, France; 5 Chaire Innovation Bloc Opératoire Augmenté (BOPA), AP-HP, Institut Mines-Télécom, Université Paris-Saclay, Villejuif, France; 6 Professional Training and Learning Laboratory (EA 7529), Conservatoire National des Arts et Métiers, Paris, France; 7 DRCI, MPI Department, Methodology, Data Management and Statistics Unit, Nancy University Hospital, Nancy, France; 8 Centre hépatobiliaire, Hôpital Paul Brousse, Villejuif, France; 9 Faculté de Médecine, Université Paris-Saclay, Kremlin-Bicêtre, France

## Abstract

**Background:**

Audiovisual recording technology, referred to as the Operating Room Black Box (ORBB), is increasingly adopted in operating rooms (ORs) across several countries. It is described as beneficial, with potential to improve training, performance, and patient safety. However, its implementation raises ethical and legal concerns, such as privacy, data security, and fears of misuse. These challenges require careful consideration alongside anticipated benefits. In France, ORBB use is emerging, yet no data exist on perceptions of professionals and patients. This study aims to identify obstacles and facilitators to ORBB implementation in French ORs.

**Methods:**

An observational, prospective, multicenter study was conducted, with questionnaire responses collected overall between 13 April 2023 and 16 July 2024. Three digital questionnaires were used: a 14-item questionnaire for anesthesia professionals, a 16-item questionnaire for surgical professionals, and a 16-item questionnaire for patients. These were distributed via partner organizations, including professional societies and patient associations. Data included sociodemographic information and opinions on risks, benefits, and conditions of ORBB implementation.

**Results:**

The study included responses from 831 participants: 45.5% anesthesia professionals, 38.6% surgical professionals, and 15.9% patients. While many professionals (80.7%) recognized educational benefits, concerns about privacy (77.8%) and anxiety from constant monitoring (80.1%) were prominent. Patient perspectives were generally supportive, with 84.8% in favor, and many viewed ORBB as enhancing accountability (77.3%). Nevertheless, privacy concerns remained (66.7%).

**Conclusion:**

These results, from the first national survey of its kind in France, provide insights into stakeholder expectations and concerns. Patient and professional perspectives show both overlaps and divergences, which must be considered to guide ORBB deployment.

**Supplementary Information:**

The online version contains supplementary material available at https://doi.org/10.1186/s13037-025-00467-7.

## Introduction

In 2004, a French national survey estimated the incidence of adverse events at 6.6 per 1,000 hospitalization days, with 35% of these events deemed preventable, highlighting the need to address preventable harm [1].

Surgery is central to healthcare delivery, accounting for approximately 312.9 million procedures worldwide in 2012, and demands substantial human and technical resources [2, 3]. Despite safeguards, surgical errors remain common and are often linked to human factors (e.g., stress, fatigue), leading to delays, prolonged anesthesia, complications, longer hospital stays, or death [4, 5].

Public authorities and clinicians have introduced safety measures, such as checklists, mandatory event reporting, and morbidity reviews, but a culture of secrecy around intraoperative events limits systematic learning and corrective actions, with reporting tools remaining underused [4, 5].

To improve transparency and continuous learning, several countries have adopted continuous audio and video recording in the operating room (OR), also known as the Operating Room Black Box (ORBB), which synchronizes audiovisual and intraoperative data for post-hoc analysis [6]. Compared with checklists or ad hoc observation, ORBB offers a holistic view of both technical and non-technical aspects of team performance. Early adopters report educational and quality-improvement gains, including fewer errors, disruptions, and distractions in the OR through recognition of these issues and implementation of corrective measures [7–14]. For example, Jung et al. reported significant reductions in technical errors and communication failures after one year of ORBB use [10]. Most professionals working in ORs equipped with ORBB acknowledge its positive impact on patient safety and educational advancements [15–18]. This technology is endorsed by university practitioners to progress towards more efficient, objective, and transparent training for surgical residents. The use of ORBB also foster hopes for more standardized surgical techniques while insurers begin to implement this solution as a way to reduce litigation frequency [19, 20].

However, implementing ORBB raises ethical, legal, and regulatory challenges. Recording requires explicit, revocable consent from patients and staff and robust data protection. Key concerns include breaches of professional secrecy, privacy intrusions, perceived surveillance or hierarchical control, and potential punitive or medico-legal use [21–24]. Implementation studies, mainly in North America, recommend clear governance (purpose-limited use for education/quality improvement), predefined ownership and access rights, limited retention, and de-identification/anonymization, supported by transparent stakeholder engagement [25, 26]. However, differences in professional cultures and legal frameworks across institutions and countries mean that findings may not generalize across settings [27]. In France, rules aligned with the General Data Protection Regulation (GDPR) impose strict requirements for consent, data anonymization, and limited data retention [28].

In France, ORBB implementation is not mandated by any public authority, but interest in the technology is growing. Some surgical departments have begun experimenting with ORBB for training or research purposes. Given this emerging interest, it is crucial to carry out a thorough and inclusive evaluation of stakeholder perceptions to anticipate barriers, build consensus, and align deployment with legal and ethical expectations.

Few studies have examined practical adoption of ORBB, and even fewer have captured the views of both professionals and patients. We address this gap in France by characterizing perceived benefits, risks, and conditions for acceptable implementation among anesthesia and surgical professionals and surgical patients, to inform a feasible and legally compliant deployment strategy.

## Methodology

A three-phase, prospective, multicenter observational study was conducted, with questionnaire responses collected overall between 13 April 2023 and 16 July 2024. Each phase involved a cross-sectional survey targeting a specific population (anesthesia professionals, surgical professionals, and patients).

### Questionnaire development

Three questionnaires (14–16 items each) were developed by the study team from a common template to enable cross-group comparison. Items were predominantly closed-ended (yes/no, multiple-choice, 4-point Likert). Content covered ORBB awareness, perceived benefits and risks, and overall willingness regarding implementation. Each questionnaire was developed and validated by the scientific team (composed of scientific directors, doctoral researchers, a methodologist, and a statistician), based on the clinical experience of practitioners and on ethnographic observations and semi-structured interviews conducted to explore professionals’ perceptions of ORBB as part of a PhD in sociology. Subsequently, the distributing organizations conducted a double-masked review for learned societies and a committee review and vote for patient associations and other groups. The questionnaires are available in Supplementary Files 1, 2, and 3.

### Study population

Eligible participants were adults (≥ 18 years old) in three groups: (1) anesthesia professionals, (2) surgical professionals, and (3) surgical patients. Participation was voluntary. Recruitment was facilitated via national professional societies, hospital networks, unions, and patient advocacy groups, which distributed the survey links to their members. Using third-party organizations broadened geographic reach and preserved respondent anonymity from investigators.

### Survey distribution

In the first phase (13 April-30 May 2023), a digital questionnaire was distributed to anesthesia professionals through the French Society of Anesthesia and Intensive Care (Société Française d’Anesthésie et de Réanimation, SFAR). The 14-item questionnaire included three sociodemographic items and 11 ORBB items (awareness, perceived benefits/risks, attitudes).

Phase 2 (13 November-8 December 2023) targeted surgical patients with a 16-item questionnaire disseminated via major patient associations (“Le Lien” and “France Assos Santé”). This questionnaire included six sociodemographic items (including prior surgery/complications), one item on perceptions of the OR, and nine ORBB items (knowledge, expected benefits/risks, willingness).

The final phase (26 February-16 July 2024) targeted surgical professionals with a 16-item survey distributed through multiple channels: the national union of OR nurses (Union Nationale des Associations d’Infirmier(e)s de Bloc Opératoire Diplômé(e)s d’État), the OR nurse training school of the Assistance Publique-Hôpitaux de Paris (AP-HP, the Paris public hospital system), the National Academy of Surgery (Académie Nationale de Chirurgie), as well as through a network of surgeons at Paris-Saclay University and a medical risk insurer (Relyens). This questionnaire included five sociodemographic items and 11 ORBB items mirroring the anesthesia survey.

### Data collection

Responses were collected electronically via a secure online platform. Participants accessed the survey through a link. Responses were anonymous (no direct identifiers collected). Participants were informed about the study’s purpose, that submission of the survey implied consent, and their right to withdraw their data.

### Ethical considerations

This study falls outside the jurisdiction of the French Jardé Law (Decree n° 2016-1537) regarding research involving human subjects. Consequently, formal approval from an ethics committee was not required.

### Data analysis

Data are presented as counts and percentages. Comparisons were performed using Chi-square tests or Fisher’s exact test. Survey data were exported and analyzed using Statistical Analysis System (SAS) version 9.4 (SAS Institute, Cary, NC, USA). A p-value < 0.05 was considered statistically significant. Key results are presented in aggregate for the entire sample of professionals or patients, and major differences by subgroup are highlighted in the text.

## Results

### Professional stakeholder perspectives (surgeons, anesthesiologists, nurses)

#### Participant characteristics and awareness of ORBB

The study included 699 healthcare professionals, consisting of anesthesia professionals (71.7% of anesthesiologists) and surgical professionals (48% of surgeons). Most respondents worked in the public sector (63.2%) and had over 10 years of professional experience (60.9%). Awareness of ORBB was higher among surgical professionals (73.5%) than anesthesia professionals (56.1%), though direct hands-on experience was limited across all groups (5.6%-17.4%). There was no significant difference in awareness of ORBB between public and private sectors. Complete data are presented in Tables 1 and 2.

#### Response rate

1042 anesthesia professionals opened the questionnaire, with 378 complete responses, representing a response rate of 36.3%.

1477 surgical professionals opened the questionnaire, with 321 complete responses, representing a response rate of 21.7%.

**Table 1:** Professional sociodemographic data. Data are presented as counts, with percentages in parentheses

	All *n* = 699	Surgical professionals^a^ *n* = 321	Anesthesia professionals^b^ *n* = 378	*P*-value
	*n* (%)	*n* (%)	*n* (%)	
**Profession**				
Medical	425 (60.8)	154 (48.0)	271 (71.7)	***< 0.0001***
Paramedical	274 (39.2)	167 (52.0)	107 (28.3)	
**Business sector**				
Private	203 (29.0)	94 (29.3)	109 (28.8)	*0.642*
Public	442 (63.2)	196 (61.1)	246 (65.1)	
Others	54 (7.7)	31 (9.7)	23 (6.1)	
**Seniority**				
< 10 years	273 (39.1)	117 (36.4)	156 (41.3)	*0.193*
> 10 years	426 (60.9)	204 (63.6)	222 (58.7)	

^a^ Surgical professionals: Medical = Surgeons; Paramedical = scrub nurses

^b^ Anesthesia professionals: Medical = Anesthesiologists; Paramedical = Nurse anesthetists

**Table 2:** Awareness of ORBB by profession. Data are presented as counts, with percentages in parentheses

Awareness level	Surgical professionals		*P*-value	Anesthesia professionals		*P*-value
	Surgeons *n* = 154 n (%)	Scrub nurses *n* = 167 n (%)		Anesthesiologists *n* = 271 n (%)	Nurse anesthetists *n* = 107 n (%)	
Personal experience	23 (14.9)	29 (17.4)	***0.02***	19 (7.0)	6 (5.6)	*0.83*
Heard about it	100 (64.9)	84 (50.3)		132 (48.7)	55 (51.4)	
Never heard about it	31 (20.1)	54 (32.3)		120 (44.3)	46 (43.0)	

### Perceived benefits

#### Educational/training value, scientific research

An overwhelming majority of the professionals (75.4% for anesthesia professionals to 86.9% for surgical professionals; p-value < 0.0001) believed that ORBB could enhance education and training. More than 50% of the surgical professionals recognized potential improvements in technical skills and teamwork.

#### Improvement in practices and patient safety

Respondents expressed a favorable opinion of optimizing overall patient care through implementing ORBB. They also highlighted ORBB’s value in identifying procedural inefficiencies and potential errors such as distractions, prolonged time between surgical procedures, and poor compliance with hygiene rules.

#### Operational efficiency

The professionals surveyed saw ORBB as an opportunity to optimize professional practices and quality of care provided, improve visibility of working conditions, and enhance the climate of safety in the workplace. All the data on perceived benefits are compiled in Table 3.

**Table 3:** Perceived benefits of ORBB. Data are presented as counts, with percentages in parentheses

Positive impact on	Surgical professionals N = 321 *n* (%)	Anesthesia professionals N = 378 n (%)	*P-value*
*Education and training*	279 (86.9)	285 (75.4)	***< 0.0001***
*Technical skills*	178 (55.5)	135 (35.7)	***< 0.0001***
*Teamwork*	166 (51.7)	177 (46.8)	*0.1977*
*Non-technical skills*	241 (75.1)	283 (74.9)	*0.9490*
*Scientific research*	257 (80.1)	244 (64.6)	***< 0.0001***
*Distraction*	275 (85.7)	320 (84.7)	*0.7074*
*Time between surgical procedures*	168 (52.3)	144 (38.1)	***0.0002***
*Compliance with hygiene rules*	207 (64.5)	266 (70.4)	*0.0974*
*Overall patient care*	198 (61.7)	205 (54.2)	***0.0470***
*Safer patient care*	218 (67.9)	201 (53.2)	***< 0.0001***
*Identification of procedural inefficiencies and potential errors*	174 (54.2)	162 (42.9)	***0.0028***
*Professional practices and quality of care*	267 (83.2)	263 (69.6)	***< 0.0001***
*Visibility of working conditions*	263 (81.9)	268 (70.9)	***0.0007***
*Climate of safety in the workplace*	164 (51.1)	159 (42.1)	***0.0171***

#### Profession comparisons

Across positive responses, paramedical professions were significantly more favorable to ORBB than medical professions (see Table 4).

**Table 4:** Perceived benefits of ORBB by profession. Data are presented as counts, with percentages in parentheses

	Training *n* (%)	Optimizing professional practices *n* (%)	Improving visibility of working conditions *n* (%)
**Nurse anesthetists**	90 (84.1)	71 (66.4)	87 (81.3)
**Anesthesiologists**	195 (72)	148 (54.6)	181 (66.8)
*P-value*	***0.01***	***0.03***	***< 0.01***
**Scrub nurses**	155 (92.8)	137 (82)	150 (89.8)
**Surgeons**	124 (80.5)	101 (65.6)	113 (73.4)
*P-value*	***< 0.01***	***< 0.001***	***0.0001***

### Perceived concerns

#### Stress, anxiety, and psychological impacts

The professionals expressed real concern about implementing ORBB. For 78.5% of surgical professionals and 81.5% of anesthesia professionals, ORBB would be a source of increased stress for professionals and patients. Use for video surveillance and hierarchical control was also described by a large proportion of respondents (81.3% and 83.3% respectively), along with a loss of autonomy and freedom in practice (71.3% and 73.3% respectively). Finally, the fear of an alteration in colleague relationships emerged for 69.8% of surgical professionals and 68.3% of anesthesia professionals.

#### Legal and ethical issues

The surgical and anesthesia professionals also expressed concern about the ethical and legal aspects of ORBB, such as the risks of breaching professional secrecy (77.3% and 78.3% respectively) and invading patient privacy (81.6% and 83.6% respectively).

Finally, the medico-legal fear that ORBB data could be used against professionals is widely expressed (78.2% and 76.2% respectively).

It should be noted that for 53% of surgical teams responding, the risks expressed were avoidable with the implementation of prior measures, compared with 38.4% of anesthesia teams responding *(*p-value *=* 0.0001*).*

All data on perceived concerns are presented in Table 5.

**Table 5:** Perceived concerns about ORBB by profession. Data are presented as counts, with percentages in parentheses

Negative impact on	Surgical professionals *n* (%)		*P*-value	Anesthesia professionals *n* (%)		*P*-value
	Surgeons	Scrub nurses		Anesthesiologists	Nurse anesthetists	
*Increase in stress for professionals and patients*	116 (75.3)	136 (81.4)	*0.1829*	225 (83.0)	83 (77.6)	*0.2186*
*Use for video surveillance and hierarchical control*	130 (84.4)	131 (78.4)	*0.1713*	234 (86.3)	81 (75.7)	***0.0124***
*Loss of autonomy and freedom in practice*	109 (70.8)	120 (71.9)	*0.8312*	208 (76.8)	69 (64.5)	***0.0152***
*Alteration in colleague relations*	113 (73.4)	111 (66.5)	*0.1780*	188 (69.4)	70 (65.4)	*0.4571*
*Breach of professional secrecy*	112 (72.7)	136 (81.4)	*0.0629*	221 (81.5)	75 (70.1)	***0.0149***
*Invasion of patient privacy*	118 (76.6)	144 (86.2)	***0.0265***	235 (86.7)	81 (75.7)	***0.0092***

### Legislative framework and organizational considerations

#### Consent, data ownership, and usage

Surgical and anesthesia professionals agreed on the importance of formalizing informed consent processes for both patients and staff (95.6% and 95.5% respectively). They also expressed a strong consensus on defining data ownership and clear guidelines for permissible uses, be it training, quality improvement, or medico-legal documentation (97.5% and 98.4% respectively).

Preparation sessions and briefings were widely supported as a means to ensure stakeholders understand the technical, legal, and ethical implications of ORBB (94.1% and 91.8% respectively, p-value *=* 0.2433). Across all professions, there was an agreement that such meetings would help mitigate confusion, clarify roles, and reinforce best practices for data handling.

### Feelings about working in an OR equipped with ORBB

Respondents were asked how they felt about working in an OR equipped with ORBB technology. The majority of respondents expressed mistrust (53.6% for surgical teams and 62.7% for anesthesia teams*).* Data on professional feelings are presented in Table 6.

**Table 6:** Respondents’ feelings about working in an OR equipped with ORBB technology. Data are presented as counts, with percentages in parentheses

Feelings	Surgical professionals *n* (%)	Anesthesia professionals *n* (%)	*P-value*
Enthusiastic	27 (8.4)	18 (4.8)	
Rather enthusiastic	51 (15.9)	61 (16.1)	
Indifferent	71 (22.1)	62 (16.4)	***0.0006***
Rather mistrustful	111 (34.6)	114 (30.2)	
Very mistrustful	61 (19.0)	123 (32.5)	

### Patient perspectives

#### Sociodemographic data

Responses were collected from 132 patients, of whom 64.4% identified as women, 34.8% as men, and 0.8% as non-binary. A significant majority, 83.3%, were aged 51 or older, with 48.5% in the 51–70 age group and 34.8% over 71 years old. In terms of education, 32.6% of respondents had only completed a baccalaureate or equivalent, while 53.8% held a bachelor’s or postgraduate degree.

Patients demonstrated an overall positive perception of the OR, associating it with terms like “organization” for 63.6%, “rigor” for 56.1%, “safety” for 44.7%, and “control” for 49.2%. However, patients also linked it to “stress” for 47.7% or “fear” for 15.2%. This dichotomy suggests that while patients recognize the structured and secure nature of the OR, a significant portion still experiences anxiety.

A notable finding was that 35.6% of responding patients reported having experienced surgical or anesthetic complications. Among these individuals, 78.7% stated that these complications had a significant impact on their daily life, with 56.8% considering these effects to be permanent.

An overwhelming majority (84.8%) supported the implementation of ORBB, indicating a general openness to technological advancements in healthcare. Patients cited reasons such as increased accountability and improved safety standards.

#### Perceived benefits

Patients primarily viewed ORBB as a tool to enhance professional accountability (77.3%), improve training (54.5%), and increase attention to patient care (68.2%). The potential for using recordings as legal evidence was a key factor. Patients with a history of complications were significantly more likely to endorse this use compared to those without (70.2% vs. 47.1%, p-value *=* 0.01).

#### Perceived concerns

However, significant concerns about patient privacy (66.7%) and anonymity (62.1%) were prevalent. Furthermore, compared with healthcare professionals, patients did not expect direct personal benefits. Few believed ORBB would reduce their anxiety (10.6%, p-value *<* 0.01*)* or increase their satisfaction with care (19.7%, p-value *<* 0.0001).

#### Legislative framework and organizational considerations

Echoing the sentiments of healthcare professionals, patients strongly endorsed the need for a clear organizational framework. A large majority called for formal consent processes for all parties (86.4%, p-value *<* 0.001) and predefined rules for data ownership and access (77.3%, p-value *<* 0.0001).

## Discussion

This first nationwide French survey on ORBB reveals broad but conditional support from key stakeholders. Both healthcare professionals and patients recognize ORBB’s potential to improve training and patient safety. However, this optimism is tempered by significant professional concerns regarding surveillance and medico-legal risks, which contrast with patients’ strong desire for accountability.

Professional perception is marked by a deep ambivalence. While a majority acknowledged benefits for education and identifying errors, these views were paired with widespread fears of increased stress, loss of autonomy, and use of recordings for punitive control. These concerns, however, varied distinctly by professional group. Surgical professionals, for instance, were most preoccupied with potential medico-legal uses of recorded data, while the anesthesia team expressed greater concern for the potential deterioration of the caregiver-patient relationship. Nurses, conversely, appeared less worried than surgeons about regulatory and legal implications but expressed stronger apprehensions about the effects of continuous surveillance on patient privacy and professional stress. This complex landscape of views was further compounded by inconsistent awareness of the technology, particularly among anesthesia professionals, highlighting a need for targeted education.

In contrast, patients demonstrated overwhelming support (84.8%), driven by a desire for transparency and accountability. This was particularly pronounced among patients who had previously experienced surgical complications and viewed ORBB as a tool for objective post-hoc analysis in the event of legal recourse. While patients shared professionals’ concerns about privacy, their priorities were clearly focused on systemic safety improvements rather than personal comfort. These findings echo those of Gallant et al. (2022), who reported that patients generally believed OR recording would enhance surgical quality and, in many cases, expressed a desire to access the recordings themselves [21].

Crucially, our study identifies a clear path forward by revealing a strong consensus on the prerequisites for implementation. Professionals and patients alike overwhelmingly agreed on the need for a robust framework built on three pillars: explicit informed consent for all parties, clear data governance defining ownership and use, and comprehensive stakeholder training. This aligns with international recommendations emphasizing transparent engagement to build trust and ensure ethical adoption. For France, this consensus is particularly critical as it reflects both ethical expectations and strict legal requirements under regulations like GDPR and national data protection laws, which are enforced by the Commission Nationale de l’Informatique et des Libertés (CNIL), the French data protection authority. The strong insistence on consent in our survey, therefore, is not merely a preference but a foundational legal mandate. This conditional acceptance, a “yes, if…”, provides a practical roadmap for institutions. To assist readers who would like to develop the system in their healthcare facility, we have created a checklist to guide them through the process (Supplementary File 4).

Our study has several limitations. First, the data are subject to response bias, as participants may hold stronger opinions than non-respondents. Second, perceptions were based on a hypothetical context, as few respondents had direct experience with ORBB; attitudes may evolve with real-world use. Third, the patient sample was skewed towards older, more educated individuals who had experienced complications, which may overstate the general patient population’s high level of support. Fourth, as multiple channels were used to contact respondents, despite the precautions taken and the high degree of response heterogeneity, we cannot guarantee with absolute certainty that there are no duplicates in the surgeon category. Finally, subgroup analyses were exploratory and not powered for definitive conclusions.

Despite these limitations, our findings place ORBB implementation in France at a pivotal moment. Successful deployment now hinges on translating stakeholder expectations into concrete institutional policies. We recommend that hospitals form multidisciplinary steering committees, including clinicians, legal experts, and patient representatives, to co-develop protocols and guide pilot projects. Future research should shift from perception to practice, using longitudinal studies to evaluate ORBB’s real-world impact on clinical outcomes, safety culture, and cost-effectiveness. By meeting the clear conditions set forth by both professionals and patients, French healthcare institutions can harness ORBB as a powerful tool for continuous improvement, advancing surgical care while preserving the essential trust of all involved.

## Supplementary Information

Below is the link to the electronic supplementary material.

Supplementary Material 1

Supplementary Material 2

Supplementary Material 3

Supplementary Material 4

## Data Availability

The datasets generated and analyzed during the current study are not publicly available due to participant privacy and confidentiality concerns but are available from the corresponding author on reasonable request.
