# Correction to: Implementation of audiovisual recording in the operating room: a nationwide survey of stakeholder perspectives in France

**DOI:** 10.1186/s13037-026-00507-w

**Published:** 2026-10-05

**Authors:** Côme Slosse, Arnaud Allemang--Trivalle, Nicolas El Haïk-Wagner, Amandine Luc, Elodie Jeanbert, Eric Vibert, Hervé Bouaziz

**Affiliations:** 1 Department of Anesthesiology and Intensive Care, University Hospital of Nancy, Nancy, France; 2 Inserm IADI U1254, University of Lorraine, Nancy, France; 3 Haptic Intelligence Department, Max Planck Institute for Intelligent Systems, Heisenbergstraße 3, 70569 Stuttgart, Germany; 4 Department of Computer Science, IMT Atlantique, Lab-STICC, UMR CNRS 6285, Brest, France; 5 Chaire Innovation Bloc Opératoire Augmenté (BOPA), AP-HP, Institut Mines-Télécom, Université Paris-Saclay, Villejuif, France; 6 Professional Training and Learning Laboratory (EA 7529), Conservatoire National des Arts et Métiers, Paris, France; 7 DRCI, MPI Department, Methodology, Data Management and Statistics Unit,, Nancy University Hospital, Nancy, France; 8 Centre hépatobiliaire, Hôpital Paul Brousse, Villejuif, France; 9 Faculté de Médecine, Université Paris-Saclay, Kremlin-Bicêtre, France

**Correction to: Patient Safety in Surgery (2025) 20:7**

**https://doi.org/10.1186/s13037-025-00467-7**

Following publication of the original article [1], we have been notified that there are several mistakes in article body text, specifically in Table 3.

It is not as follows:

Positive impact on Surgical professionals N = 321 n (%) Anesthesia professionals N = 378 n (%) P-value

Education and training 279 (86.9) 279 (86.9) < 0.0001

Technical skills 178 (55.5) 178 (55.5) < 0.0001

It should be:

Positive impact on Surgical professionals *N* = 321 n (%) Anesthesia professionals *N* = 378 n (%) P-value

Education and training 279 (86.9) 285 (75.4) < 0.0001

Technical skills 178 (55.5) 135 (35.7) < 0.0001.

The original article was updated.
