# Supplementary material for: Implementation of audiovisual recording in the operating room: a nationwide survey of stakeholder perspectives in France
Source: Patient Saf Surg. 2026 Feb 3;20:7. doi: 10.1186/s13037-025-00467-7 (PMC12870878; doi:10.1186/s13037-025-00467-7)
Supplement: Supplementary file 1 — Supplementary Material 1 [file 13037_2025_467_MOESM1_ESM.docx]

**Supplementary File 1 – Questionnaire for Anesthesia Professionals (French & English Versions)**

**Original Survey (French)**

###### Étude EVIDENCE

L’arrivée de nouvelles technologies a considérablement fait évoluer nos modes de vie et nos pratiques professionnelles.

L’enregistrement vidéo au bloc opératoire consiste à enregistrer l’image et le son de la salle d’opération en continu grâce à des caméras fixes en plan large.

Cette enquête a pour objectif de connaître l’avis des professionnels de santé (IADE et Médecins Anesthésistes-Réanimateurs) sur une pratique déjà utilisée dans certains pays (Corée du Sud, Canada et États-Unis) et qui pourrait un jour faire son apparition en France.

Répondre à ce questionnaire vous prendra environ 5 minutes.

###### Vous êtes : (Cochez la bonne réponse)

- - - - - Infirmier(e) Anesthésiste Diplômé d’État
        - Médecin Anesthésiste Réanimateur

###### Dans quel secteur travaillez-vous ? *(Cochez la bonne réponse)*

- - - - - En secteur public
        - En libéral / secteur privé
        - Dans un service de santé des armées (SSA)
        - Dans un établissement de santé privé d'intérêt collectif (ancien PSPH)

###### Quelle est votre ancienneté dans la profession *? (Cochez la bonne réponse)*

- - - - - 0 à 5 ans
        - 5 à 10 ans
        - 10 à 20 ans
        - Plus de 20 ans

###### Aviez-vous déjà entendu parler de la possibilité d’enregistrer et de filmer l’activité des soignants (chirurgiens, anesthésistes-réanimateurs et personnels paramédicaux) au bloc opératoire ? *(Cochez la bonne réponse)*

- - - - - Oui, j’en ai entendu parler
        - Oui, je l’ai même vécu
        - Non, je n’en ai jamais entendu parler

###### Parmi les propositions suivantes, quelles sont, selon vous, celles qui seront impactées par l’enregistrement vidéo au bloc opératoire ? (*Réponses possibles : D’accord, plutôt d’accord, plutôt pas d’accord, pas d’accord)*

- - - - - Les distractions au bloc opératoire (conversation privée, appel téléphonique non essentiel, bruit de fond, ouverture de portes…)
        - Les compétences techniques (pose de cathéter, intubation, …)
        - Les compétences non techniques (relation, communication, leadership, …)
        - Le délai entre deux interventions
        - L’optimisation de la prise en charge des patients
        - Le respect de l’hygiène et de l’asepsie par les intervenants

###### Merci de donner votre avis sur les assertions suivantes concernant les mesures préalables à l’installation de dispositifs permettant l’enregistrement vidéo au bloc opératoire *(Réponses possibles : D’accord, plutôt d’accord, plutôt pas d’accord, pas d’accord)*

- - - - - Organiser des réunions préparatoires et/ou des groupes de travail dédiés avant la mise en place
        - Obtenir le consentement du patient et des professionnels concernés
        - Définir à qui appartiennent les enregistrements (patients, établissements, soignants)
        - Définir l’utilisation qui sera faite de ces données (les confier au patient pour son information, les utiliser dans un but éducatif, les mettre à disposition des parties en cas de litige, …)

###### Merci de bien vouloir donner votre opinion sur les assertions suivantes concernant les intérêts potentiels de l’enregistrement vidéo au bloc opératoire pour les professionnels de santé : *(Réponses possibles : D’accord, plutôt d’accord, plutôt pas d’accord, pas d’accord)*

- - - - - Intérêt Pédagogique
        - Amélioration des pratiques professionnelles et renforcement du contrôle sur la qualité des soins
        - Amélioration des relations entre les professionnels du bloc opératoire
        - Optimisation des pratiques des professionnels de santé
        - Diminution de la fréquence d'évènements indésirables
        - Facilitation de la déclaration des évènements indésirables
        - Renforcement du climat de sécurité au sein de la salle d’opération
        - Meilleure visibilité de leur condition de travail

###### Merci de bien vouloir nous donner votre opinion sur les assertions suivantes concernant les avantages potentiels de l’enregistrement vidéo au bloc opératoire pour le patient : *(Réponses possibles : D’accord, plutôt d’accord, plutôt pas d’accord, pas d’accord)*

- - - - - Sécurisation de sa prise en charge
        - Réduction de son niveau d’anxiété
        - Augmentation du niveau de satisfaction patient

###### Merci de donner votre avis sur les assertions suivantes concernant les risques consécutifs à la mise en œuvre de l’enregistrement vidéo au bloc opératoire : *(Réponses possibles : D’accord, plutôt d’accord, plutôt pas d’accord, pas d’accord)*

- - - - - Violation du secret professionnel
        - Atteinte à l’intimité des patients
        - Augmentation du stress des professionnels de santé et des patients
        - Altération de la relation soignant-soigné dans la prise en charge des patients
        - Utilisation à des fins de vidéosurveillance et de contrôle
        - Perte d’autonomie/liberté dans la pratique et l’attitude des professionnels de santé

###### Pensez-vous que ces risques soient :

- - - - - Évitables par la mise en place de mesures préventives (création d’une charte, instauration d’un règlement, …)
        - Inévitables quelles que soient les mesures mises en place

###### Selon vous, comment les données issues de l’enregistrement vidéo et audio au bloc opératoire peuvent-elles être exploitées ? *(Réponses possibles : D’accord, plutôt d’accord, plutôt pas d’accord, pas d’accord)*

- - - - - À des fins médico-légales
        - À des fins pédagogiques
        - À des fins de recherches scientifiques
        - Pour améliorer le bien-être du patient
        - Pour donner plus de chances aux établissements de soins d’être certifiés

###### Selon vous, l’enregistrement vidéo pourrait-il avoir un impact sur le regard que vos collègues portent sur vous ou sur le regard que vous portez sur vos collègues ? *(Réponses possibles : D’accord, plutôt d’accord, plutôt pas d’accord, pas d’accord)*

- - - - - D’accord
        - Plutôt d’accord
        - Plutôt pas d’accord
        - Pas d’accord

######

###### Chez les professionnels de santé, sur quel paramètre, selon vous, l’enregistrement vidéo pourrait-il avoir un impact : *(Réponses possibles : D’accord, plutôt d’accord, plutôt pas d’accord, pas d’accord)*

- - - - - Leur niveau d’anxiété
        - Leur niveau d’engagement
        - Leur sentiment de confort et de sécurité
        - Leur confiance en soi
        - Leur qualité du travail et du professionnalisme
        - Leur capacité d’auto-évaluation

###### Quel serait votre ressenti si vous deviez travailler au bloc opératoire avec un enregistrement vidéo ?

- - - - - Enthousiaste
        - Plutôt enthousiaste
        - Indifférent(e)
        - Plutôt méfiant(e)
        - Très méfiant(e)

**English Translation**

EVIDENCE Study

The advent of new technologies has significantly changed our lifestyles and professional practices.

Video recording in the operating room involves the continuous recording of images and audio using fixed wide-angle cameras.

The purpose of this survey is to gather the opinions of healthcare professionals (certified nurse anesthetists and anesthesiologists) on a practice that is already used in some countries (South Korea, Canada, and the United States) and may one day be introduced in France.

This questionnaire will take approximately 5 minutes to complete.

**1. You are:** *(Check the correct answer)*

- - - State-certified nurse anesthetist
    - Anesthesiologist-intensivist

**2. In which sector do you work?** (Check the correct answer)

- - - In the public sector
    - In private practice/the private sector
    - In a military health service
    - In a private healthcare institution of public interest (formerly PSPH)

**3. How long have you been working in this profession?** (Check the correct answer)

- - - 0 to 5 years
    - 5 to 10 years
    - 10 to 20 years
    - More than 20 years

**4. Have you ever heard of the possibility of recording and filming the activities of healthcare professionals (surgeons, anesthesiologists-intensivists, and paramedical staff) in the operating room?** (Check the correct answer)

- - - Yes, I have heard of it
    - Yes, I have even experienced it
    - No, I have never heard of it

**5. Which of the following aspects do you think will be affected by video recording in the operating room?** (Possible answers: Agree, somewhat agree, somewhat disagree, disagree)

- - - Distractions in the operating room (private conversations, non-essential phone calls, background noise, doors opening, etc.)
    - Technical skills (catheter placement, intubation, etc.)
    - Non-technical skills (relationships, communication, leadership, etc.)
    - The time between two procedures
    - Optimization of patient care
    - Compliance with hygiene and aseptic techniques by those involved

**6. Please give your opinion on the following statements concerning the measures to be taken prior to the installation of video recording devices in the operating room** (Possible answers: Agree, somewhat agree, somewhat disagree, disagree)

- - - Organize preparatory meetings and/or dedicated working groups prior to implementation
    - Obtain consent from the patient and relevant professionals
    - Define who owns the recordings (patients, institutions, caregivers)
    - Define how the data will be used (give it to the patient for their information, use it for educational purposes, make it available to the parties in the event of a dispute, etc.)

**7. Please give your opinion on the following statements concerning the potential benefits of video recording in the operating room for healthcare professionals:** (Possible answers: Agree, somewhat agree, somewhat disagree, disagree)

- - - Educational value (teaching tool)
    - Improvement of professional practices and enhanced control over the quality of care
    - Improved relations between operating room professionals
    - Optimization of healthcare professionals' practices
    - Reduction in the frequency of adverse events
    - Facilitation of adverse event reporting
    - Enhanced safety culture within the operating room
    - Greater visibility of their working conditions

**8. Please give us your opinion on the following statements concerning the potential benefits of video recording in the operating room for the patient:** (Possible answers: Agree, somewhat agree, somewhat disagree, disagree)

- - - Safer care
    - Reduction in their anxiety level
    - Increased patient satisfaction

**9. Please give your opinion on the following statements concerning the risks associated with the implementation of video recording in the operating room:** (Possible answers: Agree, somewhat agree, somewhat disagree, disagree)

- - - Breach of professional confidentiality
    - Invasion of patient privacy
    - Increased stress for healthcare professionals and patients
    - Alteration of the caregiver-patient relationship during care
    - Use for video surveillance and monitoring purposes
    - Loss of autonomy/freedom in the practice and attitude of healthcare professionals

**10.** Do you think these risks are:

- - - Avoidable through the implementation of preventive measures (creation of a charter, introduction of regulations, etc.)
    - Inevitable regardless of the measures put in place

**11. In your opinion, how can data from video and audio recordings in the operating room be used?** (Possible answers: Agree, somewhat agree, somewhat disagree, disagree)

- - - For medico-legal purposes
    - For educational purposes
    - For scientific research purposes
    - To improve patient well-being
    - To increase the likelihood that healthcare facilities are certified

**12. In your opinion, could video recording have an impact on how your colleagues view you or how you view your colleagues?** (Possible answers: Agree, somewhat agree, somewhat disagree, disagree)

- - - Agree
    - Somewhat agree
    - Somewhat disagree
    - Disagree

**13. Among healthcare professionals, in your opinion, on which aspects could video recording have an impact?** (Possible answers: Agree, somewhat agree, somewhat disagree, disagree)

- - - Their level of anxiety
    - Their level of commitment
    - Their sense of comfort and security
    - Their self-confidence
    - The quality of their work and professionalism
    - Their ability to self-assess

**14. How would you feel if you had to work in the operating room with video recording?** (Check the correct answer)

- - - Enthusiastic
    - Somewhat enthusiastic
    - Indifferent
    - Somewhat wary
    - Very wary
