# Supplementary material for: Implementation of audiovisual recording in the operating room: a nationwide survey of stakeholder perspectives in France
Source: Patient Saf Surg. 2026 Feb 3;20:7. doi: 10.1186/s13037-025-00467-7 (PMC12870878; doi:10.1186/s13037-025-00467-7)
Supplement: Supplementary file 2 — Supplementary Material 2 [file 13037_2025_467_MOESM2_ESM.docx]

**Supplementary File 2 – Questionnaire for Patients (French & English Versions)**

**Original Survey**

###### Étude EVIDENCE

###### L’arrivée de nouvelles technologies a considérablement fait évoluer nos modes de vie et nos pratiques professionnelles.

###### L’enregistrement vidéo panoramique continu au bloc opératoire consiste à enregistrer l’image et le son de la salle d’opération en continu grâce à des caméras fixes en plan large.

###### Cette enquête a pour objectif de connaître l’avis des usagers du système de santé sur une pratique déjà utilisée dans certains pays (Corée du Sud, Canada et États-Unis), et qui pourrait un jour faire son apparition en France.

###### Durée de réponse estimée : 2 à 5 minutes.

1. **Vous êtes : (Cochez la bonne réponse)**

- Femme
- Homme
- Non-Genré

1. **Quel âge avez-vous ? (Cochez la bonne réponse)**

- Entre 18 et 30 ans
- Entre 31 et 50 ans
- Entre 51 et 70 ans
- Plus de 71 ans

1. **Quel est votre niveau d’études ou équivalence ? (Cochez la bonne réponse)**

- BEPC (brevet des collèges)
- CAP / BEP
- Baccalauréat
- DEUG / BTS / DUT / DEUST
- Licence / Licence professionnelle
- Maîtrise
- Master / Diplôme d’études approfondies / Diplôme d’études supérieures spécialisées /Diplôme d’ingénieur
- Doctorat / Habilitation à diriger des recherches
- Sans diplôme

1. **Avez-vous déjà subi une ou plusieurs complication(s) en rapport avec une intervention chirurgicale ou tout autre acte invasif nécessitant ou non une anesthésie (pendant et/ou après le geste incriminé) ? (Cochez la bonne réponse)**

- Oui
- Non

1. **Si oui : a-t-elle eu un retentissement sur votre vie quotidienne ? (Cochez la bonne réponse)**

- Oui
- Non

1. **Cette complication est : (Cochez la bonne réponse)**

- Définitive
- Temporaire
- Ne sait pas

1. **Parmi les mots suivants, lesquels vous évoquent le bloc opératoire ? 
   (Cochez la ou les bonnes réponses)**

- Organisation
- Maîtrise
- Stress
- Sécurité
- Douleur
- Rigueur
- Effrayant
- Agitation
- Intimité
- Bruyant

1. **Les avions sont équipés d’enregistreurs de vol, appelés « Boites noires », permettant de récupérer l’ensemble des informations d’un trajet aérien. Selon vous, cette technologie vous parait-elle transposable au bloc opératoire ? (Cochez la bonne réponse)**

- Oui
- Non

1. **Si oui, vous pensez que cet enregistrement devrait concerner : (Cochez la bonne réponse)**

- Le son
- L'image
- Le son et l'image
- Ni le son ni l'image

1. **Pour vous, l’enregistrement de l’image et du son au bloc opératoire en continu est : (Cochez la bonne réponse)**

- Une bonne idée
- Une mauvaise idée
- Sans opinion

1. **Selon vous, les bénéfices de l’enregistrement du son et de l’image en continu au bloc opératoire pourraient être : (Cochez la ou les bonnes réponses)**

- Prendre connaissance du déroulé de l'intervention
- La réduction du niveau d'anxiété des patients
- L'augmentation du niveau de satisfaction des patients
- L'amélioration de l'attention portée aux patients par les professionnels de santé
- L'amélioration de la formation des professionnels
- L'utilisation comme preuve dans le cadre d'une plainte déposée par le patient

1. **Selon vous, avant de se faire opérer dans un bloc opératoire équipé de l’enregistrement de l’image et du son en continu, il serait impératif : (Cochez la ou les bonnes réponses)**

- D'obtenir le consentement du patient
- Que le patient en ait été informé préalablement
- Que le patient dispose d'un droit de rétractation suite à cette information
- De définir les droits d'accès et de visionnage des enregistrements
- De respecter l'anonymat des personnes qui en font la demande
- De respecter l'intimité des patients sur l'enregistrement
- Que le patient ait la possibilité d'obtenir une copie

1. **Si vous receviez une copie de l'enregistrement du son et de l’image en continu de votre intervention, qu’en feriez-vous ? (Cochez la ou les bonnes réponses)**

- Je la conserverais comme preuve en cas de recours juridique
- Je la conserverais pour prendre connaissance de mon intervention
- Je la conserverais dans le cadre de mon suivi médical
- Je la détruirais

1. **L’existence d’un enregistrement de l’image et du son en continu au bloc opératoire serait pour vous : (Cochez la bonne réponse)**

- Un argument plutôt en faveur de l'établissement de santé lors de votre choix
- Un argument plutôt en défaveur de l'établissement de santé lors de votre choix
- Ni un, ni l'autre, je ne choisirais pas l'établissement de santé en fonction de ce critère

1. **Pensez-vous que la présence de l’enregistrement du son et de l’image en continu en salle d'opération puisse changer la qualité de la relation soignants-soignés de façon :**(Cochez la bonne réponse)

- Positive : amélioration de la prise en charge globale du patient
- Négative : sentiment de méfiance des équipes soignantes se sachant surveillées
- Neutre : aucun impact

1. **Si vous étiez concernés par un événement indésirable au bloc opératoire accepteriez-vous de participer à une réunion de débriefing (Réunion de partage et d'échange, retour d’expérience) avec les équipes de bloc opératoire ?**(Cochez la bonne réponse)

- Oui
- Non

**Nous vous remercions de votre participation.**

**English Translation**

EVIDENCE Study

The advent of new technologies has significantly changed our lifestyles and professional practices.

Continuous panoramic video recording in the operating room involves the continuous recording of images and audio using fixed wide-angle cameras.

The purpose of this survey is to find out what healthcare system users think about a practice that is already in use in some countries (South Korea, Canada, and the United States) and could one day be introduced in France.

Estimated response time: 2 to 5 minutes.

1. **Who are you?** (Check the correct answer)

- Female
- Male
- Non-binary

1. **How old are you?** (Check the correct answer)

- Between 18 and 30 years old
- Between 31 and 50 years old
- Between 51 and 70 years old
- Over 71 years old

1. **What is your level of education or equivalent?** (Check the correct answer)

- BEPC (middle school diploma)
- CAP / BEP
- Baccalaureate
- DEUG / BTS / DUT / DEUST
- Bachelor's degree / Professional bachelor's degree
- Master’s degree (Maîtrise – former system)
- Master's degree / Advanced studies diploma / Specialized higher education diploma / Engineering degree
- Doctorate / Qualification to supervise research
- No degree

1. **Have you ever experienced one or more complications related to surgery or any other invasive procedure, whether or not it required anesthesia (during and/or after the procedure in question)?** (Check the correct answer)

- Yes
- No

1. **If yes: did it have an impact on your daily life?** (Check the correct answer)

- Yes
- No

1. **This complication is:** (Check the correct answer)

- Permanent
- Temporary
- Don't know

1. **Which of the following words do you associate with the operating room?** (Check the correct answer(s))

- Organization
- Control
- Stress
- Safety
- Pain
- Rigor
- Frightening
- Agitation
- Intimacy
- Noisy

1. **Airplanes are equipped with flight recorders, known as “black boxes,” which record all information relating to a flight. In your opinion, could this technology be applied to the operating room?** (Check the correct answer)

- Yes
- No

1. **If yes, do you think this recording should concern:** (Check the correct answer)

- Audio
- Image
- Both audio and image
- Neither audio nor image

1. **In your opinion, continuous image and audio recording in the operating room is:** (Check the correct answer)

- A good idea
- A bad idea
- No opinion

1. **In your opinion, the benefits of continuous audio and video recording in the operating room could be:** (Check the correct answer(s))

- Reviewing how the procedure unfolded
- Reducing patient anxiety
- Increasing patient satisfaction
- Improving the attention given to patients by healthcare professionals
- Improving professional training
- Using the recordings as evidence in the event of a complaint filed by the patient

1. **In your opinion, before undergoing surgery in an operating room equipped with continuous image and sound recording, it would be essential to:** (Check the correct answer(s))

- Obtain the patient's consent
- Inform the patient in advance
- Give the patient the right to withdraw consent after being informed
- Define the rights of access and viewing of the recordings
- Respect the anonymity of those who request it
- Respect the privacy of patients on the recording
- Give the patient the option of obtaining a copy

1. **If you received a copy of the continuous audio and video recording of your procedure, what would you do with it?** (Check the correct answer(s))

- I would keep it as evidence in case of legal action
- I would keep it to review my procedure
- I would keep it as part of my medical follow-up
- I would destroy it

1. **Would the existence of continuous image and audio recording in the operating room be for you:** (Check the correct answer)

- An argument in favor of the healthcare facility when making your choice
- An argument against the healthcare facility when making your choice
- Neither one nor the other; I would not choose a healthcare facility based on this criterion

1. **Do you think that continuous audio and video recording in the operating room could change the quality of the caregiver-patient relationship in the following ways:** (Check the correct answer)

- Positive: improvement in overall patient care
- Negative: feeling of mistrust among healthcare teams knowing they are being monitored
- Neutral: no impact

1. **If you were involved in an adverse event in the operating room, would you agree to participate in a debriefing meeting (meeting to share and discuss experiences) with the operating room teams?** (Check the correct answer)

- Yes
- No

**Thank you for your participation.**
