# Supplementary material for: Implementation of audiovisual recording in the operating room: a nationwide survey of stakeholder perspectives in France
Source: Patient Saf Surg. 2026 Feb 3;20:7. doi: 10.1186/s13037-025-00467-7 (PMC12870878; doi:10.1186/s13037-025-00467-7)
Supplement: Supplementary file 3 — Supplementary Material 3 [file 13037_2025_467_MOESM3_ESM.docx]

**Supplementary File 3 – Questionnaire for Surgical Professionals (French & English Versions)**

**Original Survey**

Étude EVIDENCE

L’enregistrement vidéo au bloc opératoire consiste à enregistrer l’image et le son de la salle d’opération en continu grâce à des caméras fixes en plan large.

Cette enquête a pour objectif de connaître l’avis des professionnels de santé (chirurgiens et IBODE) sur une pratique déjà utilisée dans certains pays (Corée du Sud, Canada et Etats-Unis) et qui pourrait se généraliser dans notre pratique quotidienne en France.

Le temps de remplissage est estimé à 5 minutes.

Nous vous remercions par avance de vos retours.

1. **Vous êtes :** (Cochez la bonne réponse)

- Infirmier(e) de Bloc Opératoire Diplômé(e) d’Etat
- Chirurgien
- Infirmier(e) de Bloc Opératoire exerçant comme faisant fonction

1. **Dans quel secteur travaillez-vous ?** (Cochez la bonne réponse)

- En secteur public
- En libéral / secteur privé
- Dans un Service de Santé des Armées
- Dans un établissement de santé privé d'intérêt collectif (ancien PSPH)

1. **Votre statut :** (pour les chirurgiens uniquement ; veuillez sélectionner une seule des propositions suivantes)

- Interne
- Praticien hospitalier
- Praticien hospitalier avec missions de recherche
- Professeur des universités-praticien hospitalier

1. **Votre spécialité d'exercice :** (pour les chirurgiens uniquement ; veuillez sélectionner une seule des propositions suivantes)

- Chirurgie orthopédique et traumatologie
- Chirurgie thoracique et cardio-vasculaire
- Chirurgie digestive et viscérale
- Chirurgie urologique
- Chirurgie neurologique
- Chirurgie ORL et cervico-maxillo-faciale
- Chirurgie gynécologique-obstrétrique
- Chirurgie pédiatrique
- Chirurgie plastique reconstructrice et esthétique

1. **Quelle est votre ancienneté dans la profession ?** (Cochez la bonne réponse)

- > 1 an
- 1 à 3 ans
- 3 à 5 ans
- 5 à 10 ans
- 10 à 15 ans
- Plus de 15 ans

Chirurgien(ne)s : Nombre d’années d’activités depuis votre diplôme

Infirmier(e)s de Bloc Opératoire : Nombre d'années d'activité depuis votre entrée comme faisant fonction au bloc opératoire

1. **Aviez-vous déjà entendu parler de la possibilité d’enregistrer et de filmer l’activité des soignants (chirurgiens, anesthésistes-réanimateurs et personnels paramédicaux) au bloc opératoire ?** (Cochez la bonne réponse)

- Oui, j’en ai entendu parler
- Oui, je l’ai même vécu
- Non, je n’en ai jamais entendu parler

1. **Parmi les propositions suivantes, quelles sont, selon vous, celles qui seront impactées par l’enregistrement vidéo au bloc opératoire ?** (Réponses possibles : D’accord, plutôt d’accord, plutôt pas d’accord, pas d’accord)

- Les distractions au bloc opératoire (conversation privée, appel téléphonique non essentiel, bruit de fond, ouverture de portes…)
- Les compétences techniques (installation de la salle, réalisation du geste…)
- Les compétences non techniques (relation, communication, leadership…)
- Le délai entre deux interventions
- L’optimisation de la prise en charge des patients
- Le respect de l’hygiène et de l’asepsie par les intervenants

1. **Merci de donner votre avis sur les assertions suivantes concernant les mesures préalables à l’installation de dispositifs permettant l’enregistrement vidéo au bloc opératoire.** (Réponses possibles : D’accord, plutôt d’accord, plutôt pas d’accord, pas d’accord)

- Organiser des réunions préparatoires et/ou des groupes de travail dédiés avant la mise en place
- Obtenir le consentement du patient et des professionnels concernés
- Définir à qui appartiennent les enregistrements (patients, établissements, soignants)
- Définir l’utilisation qui sera faite de ces données (les confier au patient pour son information, les utiliser dans un but éducatif, les mettre à disposition des parties en cas de litige…)

1. **Merci de donner votre opinion sur les assertions suivantes concernant les intérêts potentiels de l’enregistrement vidéo au bloc opératoire pour les professionnels de santé :** (Réponses possibles : D’accord, plutôt d’accord, plutôt pas d’accord, pas d’accord)

- Intérêt pédagogique (enseignement, recherche)
- Amélioration des pratiques professionnelles et renforcement du contrôle sur la qualité des soins (meilleure visualisation du champ opératoire, connaissance des metrics d’anesthésie, etc.)
- Amélioration des relations entre les professionnels du bloc opératoire
- Optimisation des pratiques des professionnels de santé
- Diminution de la fréquence d’évènements indésirables
- Facilitation de la déclaration d’évènements indésirables
- Renforcement du climat de sécurité au sein de la salle d’opération
- Reconnaissance du stress
- Meilleure visibilité de leurs conditions de travail
- Evaluation professionnelle (notation des internes, re-certification des professionnels)
- Traçabilité des données plus conforme au cadre règlementaire
- Protection en cas de contentieux médico-légal

1. **Merci de donner votre opinion sur les assertions suivantes concernant les avantages potentiels de l’enregistrement vidéo au bloc opératoire pour le patient :** (Réponses possibles : D’accord, plutôt d’accord, plutôt pas d’accord, pas d’accord)

- Sécurisation de sa prise en charge
- Réduction de son niveau d’anxiété
- Augmentation de son niveau de satisfaction

1. **Merci de donner votre avis sur les assertions suivantes concernant les risques consécutifs à la mise en œuvre de l’enregistrement vidéo au bloc opératoire :** (Réponses possibles : D’accord, plutôt d’accord, plutôt pas d’accord, pas d’accord)

- Manque d’information ou de consentement des patients et des professionnels de santé
- Violation du secret professionnel
- Atteinte à l’intimité des patients
- Augmentation du stress des professionnels de santé et des patients
- Altération de la relation soignant-soigné dans la prise en charge des patients
- Allongement des temps opératoires
- Utilisation à des fins de surveillance vidéo et de contrôle
- Perte d’autonomie/liberté dans la pratique et l’attitude des professionnels de santé
- Possible usage médico-légal des données collectées

1. **Pensez-vous que ces risques soient :** (Cochez la bonne réponse)

- Évitables par la mise en place de mesures préventives (création d’une charte, instauration d’un règlement…)
- Inévitables quelles que soit les mesures mises en place

1. **Selon vous, comment les données issues de l’enregistrement vidéo et audio au bloc opératoire peuvent-elles être exploitées ?** (Réponses possibles : D’accord, plutôt d’accord, plutôt pas d’accord, pas d’accord)

- À des fins médico-légales
- À des fins pédagogiques
- À des fins de recherches scientifiques
- Pour améliorer le bien-être du patient
- Pour donner plus de chances aux établissements de soins d’être certifiés (meilleur pilotage du bloc, sécurisation des pratiques, etc.)

1. **Selon vous, l’enregistrement vidéo pourrait-il avoir un impact sur le regard que vos collègues portent sur vous ou sur le regard que vous portez sur vos collègues ?** (Réponses possibles : D’accord, plutôt d’accord, plutôt pas d’accord, pas d’accord)

- D’accord
- Plutôt d’accord
- Plutôt pas d’accord
- Pas d’accord

1. **Chez les professionnels de santé, sur quel paramètre, selon vous, l’enregistrement vidéo pourrait-il avoir un impact :** (Réponses possibles : D’accord, plutôt d’accord, plutôt pas d’accord, pas d’accord)

- Leur niveau d’anxiété
- Leur niveau d’engagement
- Leur sentiment de confort et de sécurité
- Leur confiance en soi
- Leur qualité de travail et de professionnalisme
- Leurs interactions informelles
- Leur charge cognitive
- Leur capacité d’auto-évaluation

1. **Quel serait votre ressenti si vous deviez travailler au bloc opératoire avec un enregistrement vidéo ? (**Cochez la bonne réponse)

- Enthousiaste
- Plutôt enthousiaste
- Indifférent(e)
- Plutôt méfiant(e)
- Très méfiant(e)

**English Translation**

EVIDENCE Study

Video recording in the operating room involves continuous image and audio recording using fixed wide-angle cameras.

The purpose of this survey is to gather the opinions of healthcare professionals (surgeons and scrub nurses) on a practice that is already used in certain countries (South Korea, Canada, and the United States) and could become widespread in our daily practice in France.

This questionnaire will take 5 minutes to complete.

We thank you in advance for your feedback.

1. **You are:** (Check the correct answer)

• State-certified operating room nurse

• Surgeon

• Operating room nurse without specialized OR certification

1. **In which sector do you work? (Check the correct answer)**

• In the public sector

• In private practice/the private sector

• In the Armed Forces Health Service

• In a private healthcare institution of public interest (formerly PSPH)

1. **How long have you been in the profession?** (Check the correct answer)

• > 1 year

• 1 to 3 years

• 3 to 5 years

• 5 to 10 years

• 10 to 15 years

• More than 15 years

Surgeons: Number of years of practice since graduation
Operating room nurses: Number of years of practice since starting work in the operating room

1. **Your status:** (for surgeons only; please select only one of the following options)

• Intern

• Hospital practitioner

• Hospital practitioner with research duties

• University professor-hospital practitioner

1. **Your specialty:** (for surgeons only; please select only one of the following options)
   - Orthopedic surgery and traumatology
   - Thoracic and cardiovascular surgery
   - Digestive and visceral surgery
   - Urological surgery
   - Neurological surgery
   - ENT and craniomaxillofacial surgery
   - Gynecological and obstetric surgery
   - Pediatric surgery
   - Reconstructive and cosmetic plastic surgery
2. **Have you ever heard of the possibility of recording and filming the activities of healthcare professionals (surgeons, anesthesiologists-intensivists, and paramedical staff) in the operating room?** (Check the correct answer)

• Yes, I have heard about it

• Yes, I have even experienced it

• No, I have never heard about it

1. **Among the following statements, which ones do you think would be affected by video recording in the operating room?** (Possible answers: Agree, somewhat agree, somewhat disagree, disagree)

- Distractions in the operating room (private conversations, non-essential phone calls, background noise, doors opening, etc.)

• Technical skills (setting up the room, performing the procedure, etc.)

• Non-technical skills (relationships, communication, leadership, etc.)

• The time between two procedures

• Optimization of patient care

• Compliance with hygiene and asepsis by those involved

1. **Please give your opinion on the following statements concerning the measures to be taken prior to the installation of video recording equipment in the operating room.** (Possible answers: Agree, somewhat agree, somewhat disagree, disagree)

• Organize preparatory meetings and/or dedicated working groups prior to implementation

• Obtain consent from the patient and the professionals involved

• Define who owns the recordings (patients, institutions, caregivers)

• Define how the data will be used (give it to the patient for their information, use it for educational purposes, make it available to the parties in the event of a dispute, etc.)

1. **Please give your opinion on the following statements concerning the potential benefits of video recording in the operating room for healthcare professionals.** (Possible answers: Agree, somewhat agree, somewhat disagree, disagree)

• Educational value (teaching, research)

• Improvement of professional practices and enhanced control over the quality of care (better visualization of the surgical field, knowledge of anesthesia metrics, etc.)

• Improvement of relationships between operating room professionals

• Optimization of healthcare professionals' practices

• Reduction in the frequency of adverse events

• Facilitation of adverse event reporting

• Strengthening of the safety climate within the operating room

• Recognition of stress

• Better visibility of their working conditions

• Professional evaluation (intern grading, professional recertification)

• Data traceability more in line with the regulatory framework

• Protection in the event of medical-legal disputes

1. **Please give your opinion on the following statements regarding the potential benefits of video recording in the operating room for the patient.** (Possible answers: Agree, somewhat agree, somewhat disagree, disagree)

• Safer care

• Reduction in their anxiety level

• Increase in their level of satisfaction

1. **Please give your opinion on the following statements concerning the risks associated with the implementation of video recording in the operating room.** (Possible answers: Agree, somewhat agree, somewhat disagree, disagree)

• Lack of information or consent from patients and healthcare professionals

• Breach of professional confidentiality

• Invasion of patient privacy

• Increased stress for healthcare professionals and patients

• Alteration of the caregiver-patient relationship in patient care

• Longer operating times

• Use for video surveillance and monitoring purposes

• Loss of autonomy/freedom in the practice and attitude of healthcare professionals

• Possible medico-legal use of the data collected

1. **Do you think these risks are?** (Check the correct answer)

• Avoidable through the implementation of preventive measures (creation of a charter, introduction of regulations, etc.)

• Inevitable regardless of the measures put in place

1. **In your opinion, how can data from video and audio recordings in the operating room be used?** (Possible answers: Agree, somewhat agree, somewhat disagree, disagree)

• For medico-legal purposes

• For educational purposes

• For scientific research purposes

• To improve patient well-being

• To give healthcare facilities a better chance of being certified (better operating room management, safer practices, etc.)

1. **In your opinion, could video recording have an impact on how your colleagues view you or how you view your colleagues?** (Possible answers: Agree, somewhat agree, somewhat disagree, disagree)

• Agree

• Somewhat agree

• Somewhat disagree

• Disagree

1. **Among healthcare professionals, in your opinion, on which aspect could video recording have an impact?** (Possible answers: Agree, somewhat agree, somewhat disagree, disagree)

• Their level of anxiety

• Their level of engagement

• Their sense of comfort and safety

• Their self-confidence

• The quality of their work and professionalism

• Their informal interactions

• Their cognitive load

• Their ability to self-assess

1. **How would you feel if you had to work in the operating room with a video recording?** (Check the correct answer)

• Enthusiastic

• Somewhat enthusiastic

• Indifferent

• Somewhat suspicious

• Very suspicious
