# Supplementary material for: Implementation of audiovisual recording in the operating room: a nationwide survey of stakeholder perspectives in France
Source: Patient Saf Surg. 2026 Feb 3;20:7. doi: 10.1186/s13037-025-00467-7 (PMC12870878; doi:10.1186/s13037-025-00467-7)
Supplement: Supplementary file 4 — Supplementary Material 4 [file 13037_2025_467_MOESM4_ESM.pdf]

ORBB Implementation Checklist

TASK	DESCRIPTION	TASK MANAGER	DUE DATE	TASK STATE
PROSPECTING	Project leader identifies the conditions for deploying the ORBB (legal, technical, temporal and financial).			
CREATING TASK FORCES	Project leader contacts the various stakeholders, including patient associations, to initiate internal discussions.			
	Creation and validation of the patient task force			
	Creation and validation of the anesthesia task force (medical and paramedical staff)			
	Creation and validation of the surgery task force (medical and paramedical staff)			
	Creation and validation of the administrative task force			
	Creation and validation of the IT task force			
	Creation and validation of the legal task force			
	Creation and validation of the financial task force			
	Creation and validation of the technical task force			
TASK FORCE MEETINGS	Appointment/election of the chairmen and secretaries of each group			
	Meeting planning			
	Setting the agenda			
	Definition of needs, expectations and concerns			
	Drafting and approving a report			
INTER-TASK FORCE MEETINGS (Chairman and Secretary of the Task Forces)	Definition of the articles of the inter-task force : appointment/election of the chairman and secretary			
	Pooling and summarizing task force reports			
	Identification of the nature of the data collected, processed and stored.			
	Identification of the data flow and storage and of the processing operations			
	Identification of the objectives and purposes of data use			
	Identification of the roles and qualifications of stakeholders (internal and external)			
	Development of criteria for acceptability by professionals and patients (creation of meaning through scientific legitimization, the improvement of the quality of care, and the promotion of best practices)			
	Drafting and validation of the ORBB deployment pre-protocol			
INSTITUTIONAL VALIDATION	Presentation of the pre-protocol to the patient committee for adjustments and validation			
	Drafting of the final protocol			
	Validation and signing of the final protocol by the various parties involved			
	Transmission of the protocol to national authorities			
PARTNERS	Contacting and discussing with insurance companies			
	Contacting and discussing with Public Health Authorities			
	Contacting and discussing with educational partners			
	Contact and discussions with technical service providers (audio and video equipment, blurring software, AI, cloud or servers if not in-house)			
	Market research, comparison of proposals, and validation of partners and subcontractors			
	Negotiation, validation, and signing of contracts.			
IMPLEMENTATION	Definition of the schedule and conditions for deployments, including a technical diagnostic of installation feasability			
	Informing operating room teams about upcoming work (periods, temporary changes related to the work)			
	Start, progress, and completion of work			
	Test period			
	End-of-construction visit and approval			
EXPERIMENTAL PHASE	Training of professionals to work in the operating room equipped with ORBB (conditions defined according to the validated protocol)			
	Writing and conducting simulated exercises in the operating room equipped with ORBB (role-playing)			
	Creation and validation of a process with service providers for managing technical issues and reporting feedback			
	Debriefing and feedback			
APPROVAL	Final validation of the ORBB system			
	Scheduling the first patients in the ORBB operating rooms			
	Performing the first surgical procedures in the ORBB operating rooms			
	Debriefing and feedback (healthcare professionals and patients)			
MONITORING AND FOLLOW-UP	Implementation of periodic monitoring			
	Conducting medical-economic impact studies			
	Conducting qualitative impact studies (professional activities and relations, patients' experiences)			
	Adjustments and modifications as necessary			
	Remote impact assessment on quality and safety criteria			

STATE KEYS
Not Started
In Progress
Completed
Pending
Late
Needs assessment
